# Emergence of Two Different Genotypes of Bagaza Virus (BAGV) Affecting Red-Legged Partridges in Spain, in 2019 and 2021

**DOI:** 10.3390/pathogens13090724

**Published:** 2024-08-27

**Authors:** Pilar Aguilera-Sepúlveda, Belén Gómez-Martín, Montserrat Agüero, Miguel Ángel Jiménez-Clavero, Jovita Fernández-Pinero

**Affiliations:** 1 Centro de Investigación en Sanidad Animal (CISA-INIA), Consejo Superior de Investigaciones Científicas (CSIC), Carretera Algete-El Casar de Talamanca, Km. 8,1, 28130 Valdeolmos, Spainfpinero@inia.csic.es (J.F.-P.); 2 Laboratorio Central de Veterinaria (LCV), Ministry of Agriculture, Fisheries and Food (MAPA), 28110 Algete, Spain; 3 CIBER of Epidemiology and Public Health (CIBERESP), 28029 Madrid, Spain

**Keywords:** Bagaza virus, emerging genotypes, phylogenetic analysis, molecular characterization, complete genome, *Alectoris rufa*, Spain

## Abstract

Bagaza virus (BAGV) is a flavivirus that affects avian species. In Europe, it was detected for the first time in Spain in 2010, exhibiting high genetic relatedness to Israel turkey meningoencephalomyelitis virus (ITMV) isolates from Israel. After a period of epidemiological silence, BAGV re-emerged, causing important outbreaks in 2019 and 2021. This study aims to characterize the newly detected strains and to elucidate if these recent outbreaks were caused by single or different virus introductions into the country. Hence, Spanish BAGV isolates from 2019 (n = 3) and 2021 (n = 1) outbreaks, obtained from red-legged partridges in Cádiz, were sequenced and further characterized. The phylogenetic analyses showed that they belong to two different genotypes: BAGV-Genotypes 1 and 2. Isolates from 2019 belong to BAGV-Genotype 1, closely related to isolates from Senegal, where BAGV has been circulating for decades. In turn, the 2021 isolates belong to BAGV-Genotype 2, closely related to those detected in Spain in 2010. Additionally, the comparison of the viral polyproteins of several BAGV isolates from both genotypes supports and confirms the phylogenetic findings. To conclude, BAGV has been introduced into Spain on at least three independent occasions, with alternating genetic clades, thus confirming that BAGV is able to sporadically reach Southern Europe.

## 1. Introduction

Bagaza virus (*Orthoflavivirus bagazaense*, BAGV) is a flavivirus belonging to the family *Flaviviridae* and genus *Orthoflavivirus*, closely related to Israel turkey meningoencephalomyelitis virus (ITMV) and groups in the Ntaya serocomplex. As with other flaviviruses, it is mainly a mosquito-borne pathogen that affects birds (which are amplifying hosts), especially those belonging to the Phasianidae family, such as turkeys, pheasants, and partridges [1].

Bagaza virus was first isolated from *Culex* mosquitoes in Bagaza district, Central African Republic, in 1966 [2]. Since then, it has been detected in several African countries, India, the Middle East, and, more recently, in Europe [3,4,5,6,7,8,9,10,11]. Despite various countries having reported the detection of BAGV in mosquitoes, the first isolation of this virus in a vertebrate host was in Spain in 2010 [9], and then in South Africa in 2016–2017, in Himalayan monal pheasants (*Lophophorus impejanus*) [6]. On the other hand, the Israel turkey meningoencephalomyelitis virus (ITMV), detected in turkeys in Israel [12], is so genetically close to BAGV that it has been proposed that both belong to the same virus species [13].

Regarding pathogenesis, depending on the infected species, BAGV causes apathy, weakness, unresponsiveness, impaired vision, severe hemolytic process, and significant weight loss, among other disease signs [1,14,15,16]. Remarkably, BAGV can be transmitted rather efficiently by direct contact among red-legged partridges, at least under experimental conditions [1]. Mortalities of 30% in red-legged partridges [1] and 40% in grey partridges [14] have been observed upon BAGV infection, which implies an enormous impact both at socio-economic levels and in the abundance of the natural populations, as well as on the ecosystems of the Iberian Peninsula [17,18]. Based on serological detection in encephalitic patients from India during the acute phase of infection, BAGV was proposed as a zoonotic pathogen [8]; however, it was unable to infect mice under experimental conditions [1], which considerably stands against this claim.

The genome of BAGV consists of a linear, single-stranded, positive-sense RNA molecule of 10,900–11,000 nucleotides in length. This molecule encodes for a single polyprotein of 3427 amino acids, which is further processed into the following three structural proteins: capsid (C), pre-membrane (prM), and envelope (E), and the following seven non-structural proteins (NS): NS1, NS2A, NS2B, NS3, NS4A, NS4B, and NS5 [18].

In Spain, BAGV was identified for the first time in September 2010, following an unusual outbreak associated with high mortality rates in red-legged partridges (*Alectoris rufa*) and common pheasants (*Phasianus colchicus*) from Cádiz province (the southernmost province of the country) [9]. Circulation of the virus was confirmed serologically in the following seasons (2011–2012) [19]. After that, BAGV was not detected again in Spain until 2019 [10] and once again in 2021—in mosquitoes [20] and in red-legged partridges. Moreover, BAGV was reported for the first time in Portugal in September 2021 in a corn bunting (*Emberiza calandra*) and several red-legged partridges [5].

Bagaza virus is a neglected arbovirus for which little is known apart from the information provided above. This study aimed at (1) elucidating the origin of the recently re-emerged BAGV strains detected in Spain after nearly a decade of epidemiological silence, (2) establishing relationships with other circulating strains from different countries, and (3) raising knowledge about the epidemiological situation and dispersal behavior of this pathogen. For this purpose, we undertook the molecular characterization and phylogenetic analysis of four Spanish BAGV isolates, obtained from outbreaks that occurred in red-legged partridges in 2019 and 2021, in Cádiz, southern Spain. 

## 2. Materials and Methods

### 2.1. Sample Collection and Preparation

**Figure 1:**
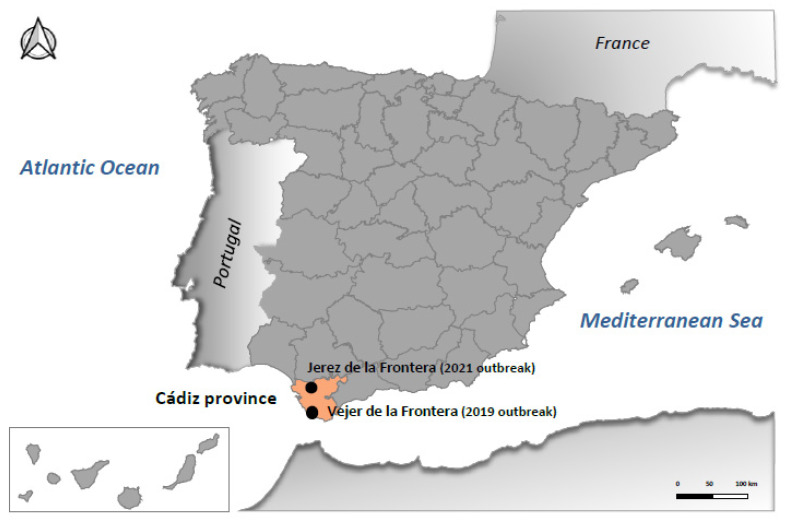
Representation of Spain highlighting Cádiz province and the municipalities where two BAGV outbreaks were reported in red-legged partridges in 2019 and 2021.

Samples included in this study were collected from four affected red-legged partridges from two independent outbreaks. The first outbreak occurred in October 2019, in Vejer de la Frontera, a municipality located in Cádiz province; thus, samples from three red-legged partridges were collected. The second outbreak took place in August 2021, in Jerez de la Frontera, Cádiz province, 70 km away from the 2019 outbreak (Figure 1); here, samples from another red-legged partridge were also collected. All samples analyzed in this study derive from brain tissues that were homogenized and subjected to initial PCR diagnostics [21] and then to virus isolation in Vero and BSR cells as formerly described [1]. Viral isolates obtained in Vero cells were further analyzed by RT-PCR for BAGV [21]. Official diagnostic techniques were carried out at the Central Veterinary Laboratory, from the Ministry of Agriculture (LCV, Algete, Spain). Then, the four isolates were stored at −80 °C and sent to the Animal Health Research Center (CISA-INIA, CSIC, Valdeolmos, Spain) for further characterization. 

### 2.2. Whole-Genome Sequencing and Phylogenetic Analyses

Full genome sequencing was carried out by overlapping conventional RT-PCRs using primer sets as previously described [9,13]. Additionally, extra primer sequences were specifically designed to cover complete viral genomes (Supplementary Table S1). Amplified products were bi-directionally sequenced by Sanger’s approach (kit Brilliant Dye Terminator Cycle Sequencing Kit version 3.1 Nimagen (Nijmegen, The Netherlands) and Thermofisher Scientific (Waltham, MA, USA)—3730 DNA Analyzer). Sequenced amplicons were further edited and assembled using SeqMan version 7.1.0 software (DNASTAR, Madison, WI, USA). Complete genome sequences obtained were submitted to GenBank. 

Phylogenetic analyses included 24 BAGV and 6 ITMV previously published complete sequences, in addition to the 4 obtained Spanish BAGV of this study. To allow the incorporation of 4 additional African isolates into this study [6], further partial phylogenetic analysis targeting 1035 Nt of the NS5 coding region was carried out, including previously employed complete sequences. Tembusu virus (MN649267) was used in both analyses as an outgroup. Multiple nucleotide sequence alignments were performed using the ClustalW algorithm within the MEGA 7 version 7.0.21 software package [22]. The best-fitting nucleotide substitution models and phylogenetic tree reconstructions were produced with dedicated applications available within the MEGA 7 software. Specifically, Maximum likelihood estimation was carried out to infer the phylogenetic trees with the highest log-likelihood value, where the evolutionary distances were computed using the optimal GTR+G+I and TN93+I models for both 35 whole-genome sequences analysis, and 39 partial-genome analyses, respectively. Bootstrap analyses were inferred from 1000 replicates.

### 2.3. Polyprotein Analysis

Complete polyprotein sequences of the four isolates were obtained by EMBOSS Sixpack tool. Sequences alignment and further amino acidic homology studies were carried out by ClustalW. Initially, the four new Spanish sequences were compared with a representative of the first outbreak of BAGV in Spain (HQ644143), used as a reference. Additionally, two sequences from Senegal 2014 (MF380434) and Portugal 2021 (LC730845) were included in this study. After that, a more complete analysis, including seventeen polyprotein sequences from several countries and years, was performed.

## 3. Results

### 3.1. BAGV Isolates

All red-legged partridge brain samples examined (*n* = 4) were positive in virus isolation after two or three passages in Vero cells. BAGV isolates analyzed by real-time RT-PCR provided Ct values ranging from 11.6 to 12.5. The names assigned to the isolates were BAGV_SPA/E/2019-01/RLP-b/3V (PP236854), BAGV_SPA/E/2019-02/RLP-b/3V (PP236853), BAGV_SPA/E/2019-03/RLP-b/3V (PP236852), and BAGV_SPA/E/2021-01/RLP-b/3V (PP236851) (Table 1). Viral isolation in BSR cells was unsuccessful.

### 3.2. BAGV Sequences and Phylogenetic Analyses

Four full genome linear sequences of 10.973 (BAGV_SPA/E/2019-01/RLP-b/3V), 10.947 (BAGV_SPA/E/2019-02/RLP-b/3V and BAGV_SPA/E/2019-03/RLP-b/3V), and 10.920 (BAGV_SPA/E/2021-01/RLP-b/3V) nucleotides were obtained. 

**Figure 2:**
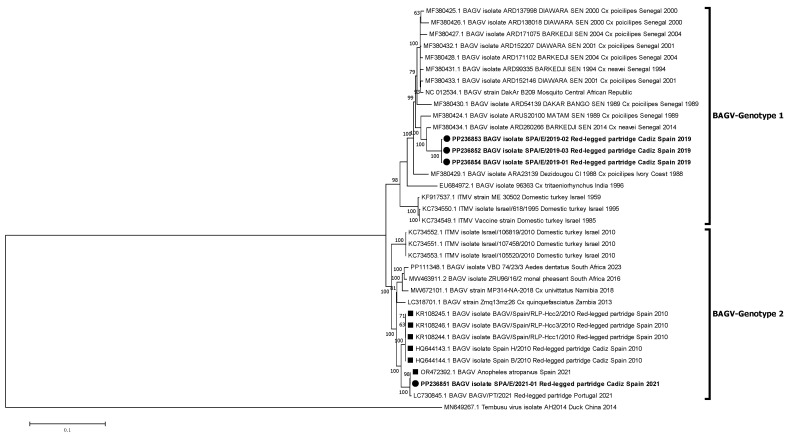
Phylogenetic analysis of 35 complete genome nucleotide sequences of BAGV/ITMV. BAGV-Genotypes 1 and 2 are indicated. Viral sequences are identified by GenBank accession number, name, species, country, and year of isolation. Sequences emphasized in bold and with a circle were generated during this study. Other Spanish strains are marked with a square. Percentages of successful bootstrap replicates over 60% are indicated at tree nodes.

**Figure 3:**
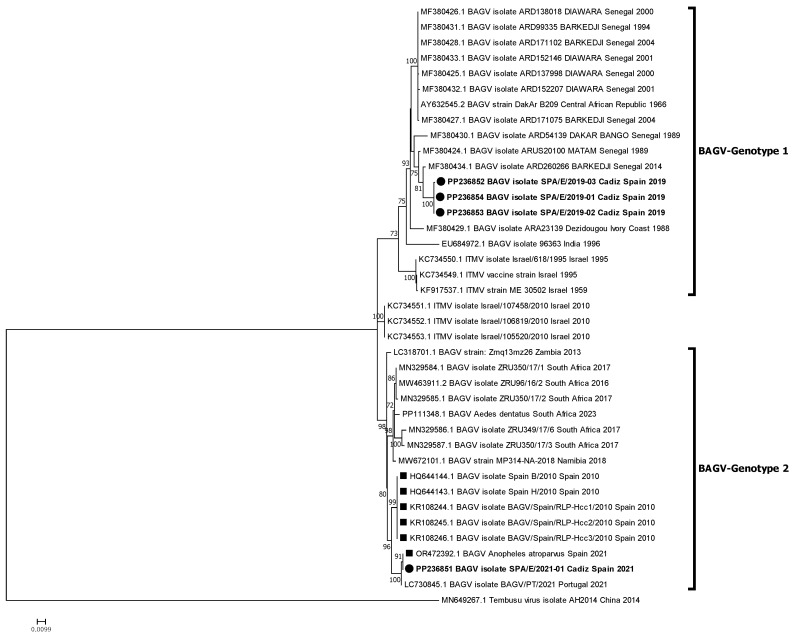
Phylogenetic analysis of 39 genome nucleotide sequences (1035 nt of NS5 region) of BAGV/ITMV. BAGV-Genotypes 1 and 2 are indicated. Viral sequences are identified by GenBank accession number, name, species, country, and year of isolation. Sequences emphasized in bold and with a circle were generated during this study. Other Spanish strains are marked with a square. Percentages of successful bootstrap replicates over 60% are indicated at tree nodes.

Phylogenetic relationships between these new isolates and other BAGV and ITMV isolates with full genome or partial (NS5) sequences available in GenBank were established. The phylogenetic analyses performed identified two genetic clusters or genotypes, here named BAGV-Genotype 1 and BAGV-Genotype 2 based on chronological order of detection. The first comprised the most ancient isolates detected (Israel, 1959–1995), as well as isolates from Senegal (1989–2014), India (1996), and Ivory Coast (1998), which clustered together with the three Spanish isolates of 2019 analyzed in this study. The second comprised more recent isolates from Israel (2010), Spain (2010), Zambia (2013), South Africa (2016, 2023), Namibia (2018), and Portugal (2021), which clustered together with the most recent BAGV identified in Spain (2021), here studied, as well as with another BAGV sequence from Spain, recently (2021) identified in *Anopheles atroparvus* mosquitoes, in Seville province, close to Cádiz [20]. Of note, both genotypes have been detected in a similar range of avian species (mainly phasianids) and mosquitoes (mainly from the *Culex* genus) (Figure 2). When comparing the sequences included in each of the two genotypes, a mean nucleotide distance estimated at 0.06996 was found, which means a 7% nucleotide divergence between the two defined genotypes. Partial phylogenetic analysis targeting 1035 Nt of the NS5 provided a similar tree topology, including the incomplete South African sequences (2017) within BAGV-Genotype 2 (Figure 3). However, the 2010 Israeli isolates did not clearly associate with any of the two main clusters, probably because of lower statistical support derived from the shorter length and lower variability in the partial NS5 sequence used in the analysis.

### 3.3. Analysis of the Viral Polyproteins

Each of the four full-length BAGV nucleotide sequences elucidated in this study yielded a polyprotein of 3427 amino acids, which were compared with those of their closest representatives from Genotypes 1 and 2, that is, Senegal 2014 and Portugal 2021, respectively (the sequence OR472392 from *A. atroparvus* mosquitoes obtained in Spain, 2021 [20], was not included in this analysis as it is incomplete in GenBank). Indeed, a representative of the initial Spanish case of BAGV (2010) was also included. In total, seven BAGV polypeptides were compared (Table 2). The 2019 Spanish isolates are characterized by a specific amino acidic signature in six positions of the polyprotein, namely, positions 108, 109, 1332, 1836, 2215, and 3372 (marked in red in Table 2). In more detail, the BAGV_SPA/E/2019-02/RLP-b/3V isolate is the only one in this study characterized by arginine (R) in position 164, instead of glutamine (G), as shown by the rest of the representatives. On the other hand, BAGV_SPA/E/2021-01/RLP-b/3V isolate has also a specific signature, particularly in positions 83, 100, 896, 1051, 2283, 2358, 2435, and 3286 of the whole polyprotein, a signature that is shared with the Portuguese isolate but differs from the initial Spanish BAGV-Genotype 2 representative (Spain 2010). When including more isolates from both clusters in this study (up to seventeen sequences), there were changes in seven amino acid positions that distinguished both BAGV genotypes (marked in red in Supplementary Table S2). The first substitution is located in glycoprotein E, position 564, while the rest of the amino acidic differences are located in non-structural proteins, in positions 1010, 1039, 1511, 1834, 2262, and 2799 (Supplementary Table S2). 

## 4. Discussion

Bagaza virus was first identified in southern Spain, in 2010 [9]. The next outbreak in Europe occurred 9 years afterwards, namely in 2019 in the same region. In 2021, another outbreak occurred in the same Spanish province, whereas BAGV was detected for the first time in Portugal in that season [5]—in an area that is close to the affected Spanish territory. Although this virus has been circulating in several African countries for at least the last three decades, it is striking that BAGV has not been detected molecularly or serologically in Spain for almost a decade since its first introduction, despite active and passive surveillance carried out in the territory. In this regard, the present study aimed to shed light on the epidemiological mechanisms by which this virus spreads between two continents through the analysis and characterization of the full-genome sequences of four BAGV isolates from Spain. The phylogenetic analyses performed identify two genetic clusters, or genotypes, of BAGV, here named BAGV-Genotype 1 and BAGV-Genotype 2, integrating strains that have been circulating in different continents (Africa, Asia, and Europe) in mosquito and avian populations. 

On the one hand, BAGV-Genotype 1 comprises more ancient and longstanding variants, including the earliest isolates of ITMV from Israel (1958–1959), which were responsible for an epizootic event that affected turkeys from that country [23]. After that, the virus continued circulating in that territory, infecting turkey flocks and becoming an emerging problem for the poultry industry because of the high economic losses it caused. To cope with this, a live-attenuated vaccine was developed [24]. This genotype was also detected in mosquitoes during an outbreak of human encephalitis in 1996 in India, which, together with serological data, suggested a zoonotic potential for this virus [8], an aspect that still remains unclear. In addition, Genotype 1 representatives were detected in *Culex* mosquito pools from West African countries, Senegal (1989–2014) and Ivory Coast (1988) [7], confirming a continued presence of this cluster in those regions. More recently, in 2019, this genotype emerged for the first time in Europe, concretely in Spain, in the same territory where the other genotype of BAGV (Genotype 2) was initially detected nine years before, in 2010.

On the other hand, BAGV-Genotype 2 seems to have evolved more recently. Members of this cluster were first identified in 2010 in Israel and Spain [9,13], affecting phasianids (turkeys, pheasants, and partridges). Later, another strain from this cluster was reported in mosquitoes (*Culex quinquefasciatus*) in Zambia in 2013. Genotype 2 strains were later identified in two southwestern African countries, particularly in *Cx. univittatus* mosquitoes from Namibia (2018) [11] and monal pheasants from South Africa (2016–2017) [6]. Another member of this genotype was also noticed in *Cx. perexiguus* from the United Arab Emirates (2018) [3] (not included in the phylogenetic analyses because of the short length of this sequence). More recently, in 2021, strains of this genotype arose simultaneously in Spain, affecting red-legged partridges and *Anopheles atroparvus* mosquitoes [20], and in Portugal, affecting wild birds [5]. Lastly, in 2023, a Genotype 2 strain was identified again in South Africa in *Aedes dentatus* mosquitoes.

These analyses confirm that there are at least two main genotypes of BAGV actively circulating for decades in bird and mosquito populations in Africa and Asia and, more recently, in Europe. In more detail, Portuguese authors propose four groups within the BAGV/ITMV monophyletic clusters, namely, G1, G2, G3, and G4, separated by low intra-genetic distances [18]. Following this proposal, BAGV-Genotype 1 would be divided into two groups as follows: G1, gathering Israeli isolates from 1959 to 1995, and G2, comprising Senegalese ones, as well as representatives from Central African Republic, India, Ivory Coast, and the Spanish isolates from 2019. On the other hand, BAGV-Genotype 2 would also be divided into two groups as follows: G3, gathering Israeli isolates from 2010, and G4, including isolates from the Iberian Peninsula, i.e., Spain, 2010 and 2021, and Portugal 2021, as well as African isolates from Namibia, Zambia, and South Africa.

These results reflect a remarkable dispersal capacity of BAGV through several territories and environments, as well as its ability to establish endemic cycles in different countries. Furthermore, they also support previous observations indicating that BAGV and ITMV likely belong to the same viral species [13,18]. Despite that, the International Committee for Viral Taxonomy (ICTV) still classifies them into two different viral species within the *Orthoflavivirus* genus (https://ictv.global/report/chapter/flaviviridae/flaviviridae/orthoflavivirus (accessed on 26 May 2024), ICTV 2023 release), a concept that is becoming obsolete as new sequence data become known. In addition, the study of the polyprotein supports the phylogenetic results, displaying each BAGV-Genotype with a specific amino acidic signature in seven positions. 

Focusing on the Spanish isolates, the emergence of BAGV in 2019 after almost a decade of silence was due to a new independent introduction of a BAGV-Genotype 1 variant from an African territory, probably related to Senegal. This connection between isolates from Senegal and Spain has also been observed in other flaviviruses such as the West Nile virus [25,26], although this observation could be biased by the origin of the available sequences. Surprisingly, two years later, in 2021, a BAGV-Genotype 2 variant reappeared in the territory after eleven years of being undetected. This re-emergence of Genotype 2 was likely caused by a new introduction/s of BAGV in the Iberian Peninsula, as judged by the highly homologous amino acid pattern that the Spanish and Portuguese 2021 isolates share in the polyprotein, suggesting a common close origin. Furthermore, this common pattern clearly differs from the original isolates from Spain (2010) as well as from other BAGV-Genotype 2 representatives. Moreover, Portuguese and Spanish 2021 isolates are not 100% homologous, neither at the nucleotide nor the amino acid level; in fact, they differ in eight amino acid positions. Consequently, the almost simultaneous detection of BAGV-Genotype 2 in neighboring Portuguese and Spanish territories might be due to either two separate introductions of the same viral strain or to a single introduction that was able to evolve and move near the border between these countries.

Hence, these findings allowed us to reconstruct the main events related to the appearance of BAGV in the Iberian Peninsula. Firstly, they suggest that there have been at least three independent introductions of BAGV in Spain. The first one occurred in 2010, caused by BAGV-Genotype 2. Then, after a silent period, a new cluster (Genotype 1) emerged in the same territory in 2019; finally, Genotype 2 re-emerged in 2021, affecting Spanish and Portuguese territories. Whether these variants are able to settle down and establish an enzootic cycle is still unknown, as more data are needed to confirm this hypothesis.

Regarding the impact of BAGV on animal health, it is an important pathogen not only for poultry farming (turkeys) but also for wild birds, particularly for some species of game birds that are raised on farms for hunting purposes, such as pheasants and partridges, supporting an economically important activity in some areas. For this reason, the presence of BAGV in red-legged partridges implies a great concern for nations such as France, Italy, Portugal, and Spain, as this is a species of high economic relevance in those countries; in fact, it is the only autochthone partridge species in Portugal [18]. Taking into account the results of this study and considering that phasianids are highly susceptible to BAGV infection, it is necessary to reinforce surveillance activities in the south of the Iberian Peninsula in order to provide an early warning and to apply the appropriate control measures to reduce the number of affected animals and subsequent economic losses. Of note, BAGV has been detected in the Iberian Peninsula only when outbreaks occur in these avian species, and only one mosquito pool was found positive in 2021 [20], despite the intense flavivirus vector surveillance carried out in southern Spain. This fact contrasts with the situation in Africa, where most BAGV data come from mosquito detections.

In summary, this study confirms the re-introduction of two BAGV variants in Spain, after a long period of epidemiological silence. As the red-legged partridge is highly susceptible to BAGV infection and disease, it would constitute a suitable target species for BAGV surveillance.

## Figures and Tables

**Table 1:** Information on the samples analyzed in this study. Results of the real-time RT-PCR, the virus isolation in cell culture, and GenBank accession no. are also presented.

Name of the Isolate	Year	Location	Species	Tissue	RRT-PCR (Ct Value) ^1^	Viral Isolation in Vero Cells, (nº of Passages, p)	GenBank Accession nº
BAGV_SPA/E/2019-01/RLP-b/3V	2019	Vejer de la Frontera (Cádiz, Spain)	Red-legged partridge	Brain	11.6	Positive (2p)	PP236854
BAGV_SPA/E/2019-02/RLP-b/3V	2019	Vejer de la Frontera (Cádiz, Spain)	Red-legged partridge	Brain	12.1	Positive (2p)	PP236853
BAGV_SPA/E/2019-03/RLP-b/3V	2019	Vejer de la Frontera (Cádiz, Spain)	Red-legged partridge	Brain	11.9	Positive (2p)	PP236852
BAGV_SPA/E/2021-01/RLP-b/3V	2021	Jerez de la Frontera (Cádiz, Spain)	Red-legged partridge	Brain	12.5	Positive (3p)	PP236851

^1^ Buitrago et al., 2012 [21].

**Table 2:** Comparison of the amino acid substitutions among the complete genomes of the Spanish BAGV isolates. The first Spanish isolate from 2010 was used as the reference sequence. The closest representatives from BAGV-Genotypes 1 and 2 (Senegal 2014 and Portugal 2021, respectively) were included in this study for comparison. Black dots indicate the same amino acid as the reference sequence. Amino acids that are different from the reference sequence (Spain 2010) are highlighted in bold. Amino acids that characterize the 2019 Spanish isolates from BAGV-Genotype 1 are marked in red.

		BAGV-GENOTYPE 2			BAGV-GENOTYPE 1			
PROTEIN	Amino Acid Position	HQ644143.1 (Spain 2010) Red-Legged Partridge	PP236851 (Spain 2021) Red-Legged Partridge	LC730845.1 (Portugal 2021) Red-Legged Partridge	PP236854 (Spain 2019) Red-Legged Partridge	PP236853 (Spain 2019) Red-Legged Partridge	PP236852 (Spain 2019) Red-Legged Partridge	MF380434.1 (Senegal 2014) *Cx. poicilipes*
**C**	**53**	**A**	**.**	**.**	**T**	**T**	**T**	**T**
	**76**	**V**	**.**	**.**	**.**	**.**	**.**	**G**
	**83**	**K**	**R**	**R**	**.**	**.**	**.**	
	**92**	**M**	**.**	**.**	**.**	**.**	**.**	**.**
	**100**	**G**	**S**	**S**	**.**	**.**	**.**	**.**
	**108**	**T**	**.**	**.**	**I**	**I**	**I**	**.**
	**109**	**L**	**V**	**V**	**T**	**T**	**T**	**S**
	**113**	**I**	**.**	**.**	**V**	**V**	**V**	**V**
	**115**	**A**	**V**	**V**	**V**	**V**	**V**	**V**
	**116**	**V**	**.**	**.**	**A**	**A**	**A**	**A**
**prM**	**150**	**A**	**.**	**.**	**T**	**T**	**T**	**T**
	**164**	**G**	**.**	**.**	**.**	**R**	**.**	**.**
	**166**	**I**	**M**	**M**	**M**	**M**	**M**	**M**
**E**	**564**	**T**	**.**	**.**	**A**	**A**	**A**	**A**
	**659**	**K**	**.**	**R**	**.**	**.**	**.**	**.**
	**699**	**S**	**.**	**F**	**.**	**.**	**.**	**.**
**NS1**	**838**	**E**	**.**	**D**	**.**	**.**	**.**	**.**
	**841**	**E**	**.**	**.**	**G**	**G**	**G**	**G**
	**842**	**K**	**.**	**.**	**R**	**R**	**R**	**R**
	**874**	**Q**	**.**	**.**	**.**	**.**	**.**	**P**
	**896**	**W**	**L**	**L**	**.**	**.**	**.**	**.**
	**909**	**G**	**.**	**.**	**.**	**.**	**.**	**E**
	**1010**	**I**	**.**	**.**	**V**	**V**	**V**	**V**
	**1051**	**K**	**R**	**R**	**.**	**.**	**.**	**.**
	**1054**	**V**	**.**	**.**	**.**	**.**	**.**	**G**
**NS2A**	**1174**	**V**	**M**	**M**	**M**	**M**	**M**	**M**
	**1202**	**L**	**.**	**M**	**.**	**.**	**.**	**.**
**NS2B**	**1305**	**R**	**.**	**.**	**K**	**K**	**K**	**K**
	**1307**	**V**	**.**	**.**	**I**	**I**	**I**	**I**
	**1319**	**V**	**.**	**.**	**I**	**I**	**I**	**I**
	**1332**	**I**	**.**	**.**	**V**	**V**	**V**	**.**
**NS3**	**1511**	**K**	**.**	**.**	**R**	**R**	**R**	**R**
	**1547**	**H**	**.**	**R**	**.**	**.**	**.**	**.**
	**1562**	**D**	**.**	**.**	**.**	**.**	**.**	**V**
	**1758**	**V**	**.**	**.**	**I**	**I**	**I**	**I**
	**1834**	**M**	**.**	**.**	**V**	**V**	**V**	**V**
	**1836**	**V**	**.**	**.**	**L**	**L**	**L**	**.**
	**1885**	**Q**	**.**	**P**	**.**	**.**	**.**	**.**
	**1887**	**N**	**.**	**Y**	**.**	**.**	**.**	**.**
	**2113**	**E**	**D**	**D**	**D**	**D**	**D**	**D**
**NS4A**	**2215**	**V**	**.**	**.**	**A**	**A**	**A**	**.**
	**2262**	**V**	**.**	**.**	**I**	**I**	**I**	**I**
	**2265**	**T**	**.**	**.**	**A**	**A**	**A**	**A**
**NS4B**	**2283**	**S**	**N**	**N**	**.**	**.**	**.**	**.**
	**2288**	**A**	**.**	**T**	**.**	**.**	**.**	**.**
	**2358**	**I**	**T**	**T**	**.**	**.**	**.**	**.**
	**2435**	**I**	**V**	**V**	**.**		**.**	**.**
	**2475**	**T**	**.**	**.**	**.**	**.**	**.**	**N**
	**2477**	**I**	**.**	**.**	**.**	**.**	**.**	**D**
	**2478**	**E**	**.**	**.**	**.**	**.**	**.**	**R**
	**2479**	**G**	**.**	**.**	**.**	**.**	**.**	**R**
	**2480**	**A**	**.**	**.**	**.**	**.**	**.**	**S**
	**2481**	**A**	**.**	**.**	**.**	**.**	**.**	**S**
	**2482**	**G**	**.**	**.**	**.**	**.**	**.**	**R**
	**2483**	**R**	**.**	**.**	**.**	**.**	**.**	**T**
	**2484**	**I**	**.**	**.**	**.**	**.**	**.**	**D**
	**2485**	**W**	**.**	**.**	**.**	**.**	**.**	**M**
	**2486**	**N**	**.**	**.**	**.**	**.**	**.**	**E**
	**2487**	**A**	**.**	**.**	**.**	**.**	**.**	**C**
	**2519**	**S**	**.**	**.**	**.**	**.**	**.**	**G**
**NS5**	**2652**	**I**	**V**	**V**	**.**	**.**	**.**	**.**
	**2698**	**T**	**.**	**.**	**.**	**.**	**.**	**I**
	**2703**	**I**	**V**	**V**	**.**	**.**	**.**	**.**
	**2799**	**N**	**.**	**.**	**K**	**K**	**K**	**K**
	**2806**	**T**	**.**	**.**	**M**	**M**	**M**	**M**
	**2895**	**A**	**.**	**.**	**S**	**S**	**S**	**S**
	**3048**	**G**	**.**	**.**	**S**	**S**	**S**	**.**
	**3286**	**G**	**S**	**S**	**.**	**.**	**.**	**.**
	**3372**	**S**	**.**	**.**	**G**	**G**	**G**	**.**

## Data Availability

The sequences presented in this study are openly available in the GenBank database.

## Supplementary Materials

The following supporting information can be downloaded at https://www.mdpi.com/article/10.3390/pathogens13090724/s1, Table S1: Sequences of primers specifically designed to complete the whole-genome sequencing of the Spanish BAGV isolates displayed in this study; Table S2: Comparison of amino acid substitutions among complete BAGV/ITMV isolates. The first Spanish isolate from 2010 was used as a reference sequence. Spanish isolates (2019 and 2021) obtained in this study were included as well as their closest representatives from BAGV-Genotypes 1 and 2 (Senegal 2014 and Portugal 2021, respectively). Additionally, more representatives from several countries and years were incorporated in this study. Black dots indicate the same amino acid as the reference sequence. Amino acids that are different from the initial reference are highlighted in bold. Amino acid changes that distinguish BAGV-Genotypes 1 and 2 are marked in red. Cell colors indicate the amino acid category: yellow = hydrophobic; green = polar; pink = acidic; blue = basic.
