# Supplementary material for: Emergence of Two Different Genotypes of Bagaza Virus (BAGV) Affecting Red-Legged Partridges in Spain, in 2019 and 2021
Source: Pathogens. 2024 Aug 27;13(9):724. doi: 10.3390/pathogens13090724 (PMC11434994; doi:10.3390/pathogens13090724)
Supplement: Supplementary file 1 [file pathogens-13-00724-s001.zip › Supplementary_Table_S1.pdf]

Supplementary Table 1. Sequences of primers specifically designed to complete the whole genome sequencing of the Spanish BAGV isolates displayed in this study.

Primer ID (ref. MF380434.1)	Sequence (5´- 3´)
**BagV-4201F**	GGATGGCCAGTGAGTGAAGCC
**BagV-4754cR**	TCGTCACATGCCACATCGTG
**BagV-4968F**	CAGTCACCCTTGATTACCC
**BagV-5649cR**	ATCCACTCATATCCTGAGTTCC
**BagV-4959F**	GGAGAAATCGGAGCAGTCAC
**BagV-5627R**	CCTGAGTTCCATGCTCTGTC
**BagV-7704F**	CCCAACTCAATCAATTAACACGG
**BagV-8239R**	CAGGACTCTTGGCATGTATGG
**BagV-8151F**	TGCAAACTGTGGATGAGTGG
**BagV- 8778R**	GCCGTGATTTTCATGACTTCTG
**BagV-8541F**	GAATTTTCCACCACATGGC
**BagV- 9168R**	CTGCTCATCCAGTGATCCTCG
**BagV-9028F**	CATGATGGGAAAGAGAGAAAAGAAAATGGGAG
**BagV-9584R**	CCAACCCTTCAACTTCTTCAGGCTTCAGC
**BagV-10549F**	GCCACCGGATGTTGGATG
**BagV-10941cR**	AGACACTGTGTTCTACCACCA
